# Pilot-scale production of a highly thermostable α-amylase enzyme from *Thermotoga petrophila* cloned into *E. coli* and its application as a desizer in textile industry

**DOI:** 10.1039/c8ra06554c

**Published:** 2019-01-08

**Authors:** Asma Zafar, Muhammad Nauman Aftab, Irfana Iqbal, Zia ud Din, Mushtaq Ahmad Saleem

**Affiliations:** Faculty of Life Sciences, University of Central Punjab Lahore Pakistan asma.zafar@ucp.edu.pk +93006485797; Institute of Industrial Biotechnology, Government College University Lahore Pakistan nauman535@yahoo.com; Department of Zoology, Lahore College for Women University Lahore Pakistan; University of Baluchistan Quetta Pakistan

## Abstract

In this study, the industrial applications of a highly thermostable α-amylase as a desizer in the textile industry was evaluated. The cloned gene was expressed in different media (ZBM, LB, ZYBM9, and ZB) with IPTG (isopropyl β-d-1-thiogalactopyranoside) used as an inducer. Lactose was also used as an alternate inducer for the T7 promoter system in *E. coli*. For the large-scale production of the enzyme, different parameters were optimized. The maximum enzyme production was achieved when the volume of medium was 70% of the total volume of fermenter with a 2.0 vvm air supply and 20% dissolved oxygen at a 200 rpm agitation rate. Under all the optimized conditions, the maximum enzyme production was 22.08 U ml^−1^ min^−1^ with lactose (200 mM) as an inducer in ZBM medium. The desizing potential of the purified α-amylase enzyme was calculated with different enzyme concentrations (50–300 U ml^−1^) at different temperatures (50–100 °C), and pHs (4–9) with varying time intervals (30–120 min). The highest desizing activity was found when 150 U ml^−1^ enzyme units were utilized at 85 °C and at 6.5 pH for 1 h.

## Abstract 2

Thermostable recombinant α-amylase was produced in a large scale for the desizing of cotton cloth in the textile industry.

## Graphical abstract

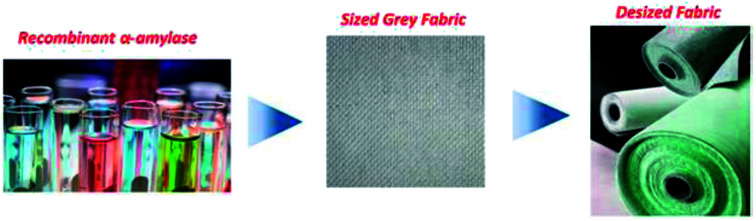

## Introduction

Amylases are starch-degrading enzymes that are capable of hydrolyzing the internal α-1-4 glycosidic linkage in starch arranged in the form of polymers composed of glucose units.^1^ These enzymes are considered to be one of the most important industrial enzyme and make up about 25% of the world enzyme market and have proven significance for biotechnology.^2,3^ These enzymes are widely distributed throughout the biodiversity and can be obtained from various sources, such as animals, plants, and microorganisms; however, the microbial amylases are generally considered the most able to meet industrial requirements due to their stability and broad range of industrial applications.^4,5^ Amylases are consequently used in many industrial applications, such as food, fermentation, textile desizing, paper, detergents, starch saccharification, and in the pharmaceutical industries.^6,7^

The thermostability of an enzyme is a desired characteristic for most industrial applications. As starch become soluble at 100 °C and above, most industrial applications of α-amylases require their use at temperatures up to 110 °C.^8^ To fulfil the industrial demands for thermostable amylases with novel properties, the excursion must be constant.^9,10^ At an industrial scale, the growth of thermophilic organisms becomes difficult due to the rigorous growth requirements.^11^ This problem has been overcome in most cases by the application of recombinant DNA technology through gene cloning into the mesophilic host.^12^

To obtain a high level of expression from the cloned genes there is a need to develop a novel process.^13^ A very efficient T7 expression system was developed into the *E. coli* strain BL21 for the heterologous expression of recombinant proteins.^14^ T7 promoter-based pET vectors are the most commonly used vectors for the expression of recombinant proteins as first reported by Studier *et al.*^15^ IPTG is mostly used as an inducer to regulate the expression of recombinant genes under the influence of a promoter. However, there is a need for an alternative inducer as IPTG is very expensive and toxic to cells.^16^ As the natural inducer of lac operon, lactose is very cheap compared to IPTG and is harmless to the host cells,^17^ and is also used as a carbon source and energy source by cells.^18^

Optimization of the culture conditions and fermentation medium to reduce the costs and to achieve maximum enzyme production was the key aim of the present research. Different fermentation techniques, including submerged fermentation and solid-state fermentation techniques, have been applied in practice. Submerged fermentation is a process of choice for many industrial applications due to various reasons, including good process control, effective bioreactor design, and it allows fermentation modeling.^19^ With respect to downstream processing, submerged fermentation has clear advantages in terms of the costs related to components involved in the medium.^20^

The large-scale production of α-amylase is attracting much research attention due to the effective development in fermentation techniques.^21^ In biotechnological processes, maximizing enzyme production is becoming one of the most important goals.^22^ The production of enzymes can be boosted by optimizing the culture conditions, like the inoculum size, dissolved oxygen level, rate of agitation, and agitation itself.^23^

In the textile industry, amylases are used in the desizing of cotton cloth. Starch is a sizing agent that is commonly used in the manufacturing process of cloth to strengthen the thread before fabric production by forming a layer on it. This layer is later removed from the woven cloth in the textile finishing industry by the application of amylase, which effectively removes the starch without damaging the fibers.^7,24^ With the introduction of mesophilic, thermostable, and alkaliphile amylases from bacterial sources enzymatic desizing is preferred due to their stability against the chemicals in the desizing liquor.^25^ Depending upon the enzyme reaction type, the temperature conditions and the mass transfer actions in the process, the degradation of starch takes the longest time among the various steps involved in desizing. Due to this reason, thermostable amylases are attracting more interest because their use can shorten the process duration to allow a similar efficiency as that of mesophilic amylases and conventional processes.^26^

This study aimed to optimize all the essential parameters in order to develop a fermentative strategy for the large-scale production of cloned α-amylase from *Thermotoga petrophila* into *E. coli* and to evaluate the desizing potential of the α-amylase.

## Methodology

### Selection of bacterial strains and plasmids

*Thermotoga petrophila* genomic DNA was purchased from DSMZ's German collection of cell cultures and microorganisms, Germany, and was used for amplification of the amylase gene. The pET 21a (+) vector was used to express the amylase gene in *E. coli* BL21.

### Cloning of the α-amylase gene from *Thermotoga petrophila* in pET-21a (+)

Amplification of the amylase gene of *Thermotoga petrophila* was carried out using genomic DNA as a template. Primers were designed from a DNA sequence retrieved from the NCBI database (GenBank accession number NC_009486.1). DNASTAR software was used for designing the primer. An *Nde*I restriction site was introduced on the 5′ end of the forward primer and a *Hind*III restriction site was introduced on the 5′ end of the reverse primer. The nucleotide sequences of the pair of primers were as follows:

Forward 5′-CATATGCTTTTGAGAGAGATAAACCGATACTGC-3′

Reverse 5′-TCACTCCTGTACAACAAGAACAAAATCAAGGGGT-3′

The PCR product obtained was analyzed on 1% agarose gel and purified using a DNA purification kit from Qiagen. The purified amplified *Thermotoga petrophila* α-amylase gene (1914 bp) was double digested with the restriction enzymes *Nde*I and *Hind*III. The digested gene was purified and ligated with double-digested pET-21a (+). Freshly prepared competent cells of *E. coli* BL21 (DE3)^27^ were used as the host organism for transformation of the recombinant vector pET 21a (+) containing the α-amylase gene. Positive clones were screened by restriction analysis of the pET 21a (+) plasmid containing the α-amylase gene with *Hind*III and *Nde*I.

### Expression of the recombinant α-amylase gene in *E. coli* BL21

For expression of the recombinant enzyme, transformed *E. coli* BL21 cells were grown in Luria–Bertani medium (0.1 liter) containing 100 μg ml^−1^ ampicillin at 37 °C, to an optical density of 0.5–0.7 at 600 nm. Then, 0.5 mM isopropyl-d-thiogalactopyranoside (IPTG) was used as an inducer and the cells were incubated at 37 °C for an additional 4 h. After 4 h incubation, the cells were harvested by centrifugation (6000 rpm, 10 min, 4 °C). The cell were lysed by sonication for 10 min after resuspension in 5 ml of 50 mM Tris–HCl (pH 7.5 to 8) and then centrifuged at 12 000 rpm for 10 min. Total protein concentration was determined using the method given by Bradford.^28^ Then, α-amylase expression was determined in the extracellular as well as in intracellular fractions by running 12% SDS-PAGE, visualized with Coomassie blue.^29^

### Fermentation techniques

#### Stirring fermenter

Fermentation was performed at 37 °C for 4 h in a glass fermenter of 7.5 L (Bioflo 110, Brunswick Scientific, USA). LB medium was autoclaved at 121 °C and 15 lb per in^2^ pressure for 30 min. Aseptic conditions were used to transfer the inoculum, which was incubated at 22 °C. The agitation and aeration rates were maintained at 200 rpm and 2.0 vvm, until the optical density reached up to 0.6. Air was sterilized using a membrane filter of 0.4 μm. For the control, frothing sterilized silicone oil (10%) was used. When the optical density reached 0.6, the cells were induced by 0.5 mM IPTG and incubated at 22 °C for 4 h. Fermented broth was centrifuged for 20 min at 6000 g and 4 °C. The supernatant was stored at 4 °C and the *E. coli* cells were sonicated. The clear cell lysate and supernatant were used for intracellular and extracellular enzyme estimation, respectively. The effects of various parameters utilized in the stirred fermenter are mentioned below:

##### 1. Effect of agitation

To determine the effect of agitation on α-amylase production in the stirred fermenter, 100–300 rpm agitation speed was utilized with a 3% inoculum size and 20% dissolved oxygen level, 60% volume of fermentation medium, and 3 vvm aeration rate.

##### 2. Effect of the inoculum

To determine the effect of the inoculum, the fermentation medium was inoculated with varying percentages (1–6%) of inoculums, with the other conditions being 200 rpm agitation rate, 20% dissolved oxygen level, 60% volume of fermentation medium, and 3 vvm aeration rate.

##### 3. Effect of dissolved oxygen

Various dissolved oxygen levels, ranging from 10–30%, were analyzed by providing 3% inoculum at 200 rpm agitation speed and 3 vvm aeration rate using 60% volume of fermentation medium in order to obtain the maximum enzyme production.

##### 4. Effect of the volume of the fermentation medium

To determine the effect of the volume of the fermentation medium for achieving the maximum enzyme production, different volumes from 40–90% of the fermentation medium were used in a stirred fermenter, with the other conditions being 3% inoculum size, 200 rpm agitation speed, 20% dissolved oxygen level, and 3 vvm aeration rate.

##### 5. Effect of the aeration rate

The effect of varying the rate of aeration on the production of recombinant α-amylase was studied at different aeration levels (0.5–3.0 vvm), with the other conditions being 3% inoculum size, 200 rpm agitation rate, 20% dissolved oxygen level, and 70% volume of the medium.

#### Enzyme assay

Enzyme activity was analyzed using the DNS (dinitrosalicylic acid) method^30^ by determination of the reducing sugars. Maltose was used as a standard to measure the liberated reducing sugars in a spectrophotometer at 550 nm. Enzyme activity was defined as “the quantity of enzyme that was used to release reducing sugar (one μmole) from a substrate under specific conditions”.

#### Protein assay

The Bradford method^28^ was used for estimation of the total proteins using bovine serum albumin as standard. Here, 0.2 ml of enzyme and 5.0 ml of Bradford reagent were mixed at room temperature and the absorbance was measured after 5 min at 595 nm in a spectrophotometer. A blank (without enzyme) was run in parallel containing distilled water instead of the enzyme.

### Media optimization with lactose and IPTG as the inducer

Different media (M9, LB, ZB, ZBM, and ZYBM9) were optimized to get the maximum production of recombinant α-amylase by utilizing lactose and the IPTG alternate inducer for the T7 expression system.

### Industrial applications of the recombinant α-amylase

#### Desizing with alpha amylase

To check the desizing activity of the recombinant enzyme, a piece of grey fabric was used having maximum starch on it. A plain weaved, 100% cotton grey fabric was used in this experiment. The sized fabric was dipped in boiling water (100 °C) for almost 2 min to facilitate the swelling of the starch layer. A fabric piece (5 × 5 inches) was pre-weighed and used for further analysis. The alignment of the warps and wefts in the plain weaved fabric was done a simple crisscross pattern^31^ that is used more than any other weave. Plain weaved fabric is stronger than any other weave type due to the presence of a greater number of intersections per unit space, all other factors being equal.^32^ The pre-weight of the cloth having starch on it was calculated to be 13.78 g. Total fiber count was 120 epi in the grey fabric used in this study. The fabric was dipped in α-amylase solution (pH 7.0, 100 ml) and then incubated at 80 °C for 1.0 h. After 1 h incubation, washing was carried out with distilled water and the fabric strip was then dried in an oven. After complete drying, the strip was weighed again.Weight of starch removed = initial weight − final weight.

The % removal of starch was calculated by applying the following formula:
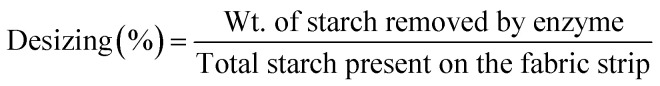


The total starch present in the grey fabric was calculated by hydrolyzing the starch with H_2_SO_4_ (0.1 N).^33–38^

#### Optimization of the cotton cloth desizing

The effect of different parameters, such as pH (4–9), temperature of incubation (50–100 °C), enzyme concentration (50–300 U ml^−1^ min^−1^), time of incubation (30–120 min), effect of agitation rate (50–250 rpm), effect of various metal ions (10 mM) (Mg^2+^, Cu^2+^, Ca^2+^, Co^2+^, Na^1+^, K^1+^, NH_4_^1+^, and Ni^2+^) at a concentration of 10 mM, and effect of different wetting agents (SDS, Tween 20, Tween 80, Triton X-100, CTAB, CABP) at a concentration of 10 mM were studied to investigate the maximum desizing potential of the recombinant α-amylase for use in the textile industry. The desizing efficiency of the recombinant α-amylase enzyme was determined by iodine solution test.

## Results and discussion

Thermostable enzymes are required for all industrial processes that are usually carried out at very high temperature.^39^ The textile industry uses crude amylase for the desizing of grey fabric as a very auspicious process in cloth processing.^35^

### Cloning of amylase gene in the expression vector pET-21a (+)

**Fig. 1:**
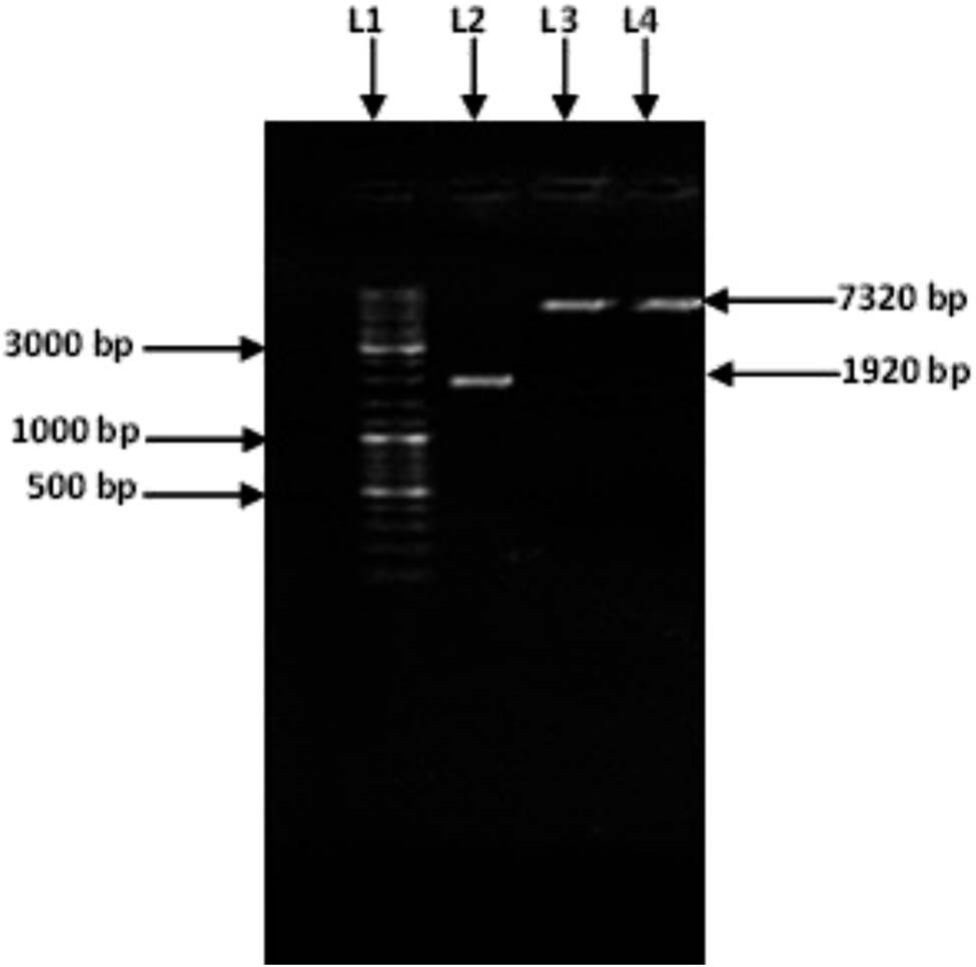
Amplified amylase gene: the GeneRuler™ DNA ladder (Lane 1), PCR product of the amylase gene 1920 bp (Lane 2), single digested recombinant pET 21a (+) containing the amylase gene (Lanes 3 and 4).

Amplification of an amylase gene from *T. petrophila* was carried out using a specific pair of primers. A specific amplified band (1920 bp) (Fig. 1) was purified and double digested using the restriction enzymes *Nde*I and *Hind*III. The purified double-digested and amplified amylase gene was ligated into the pET21a (+) vector with the help of T4 DNA ligase. The recombinant vector pET21a (+)/amylase, was introduced into the freshly prepared competent cells of *E. coli* BL21 (DE3). Cloning of the amylase gene was confirmed by double digesting it with *Hind*III and *Nde*I and by sequencing the clone gene. After a single digestion of recombinant plasmid with *Hind*III, a band of 7320 bp was observed (Fig. 1) confirming the cloning of amylase gene in the pET21a (+) vector.

### Expression of the cloned amylase gene

**Fig. 2:**
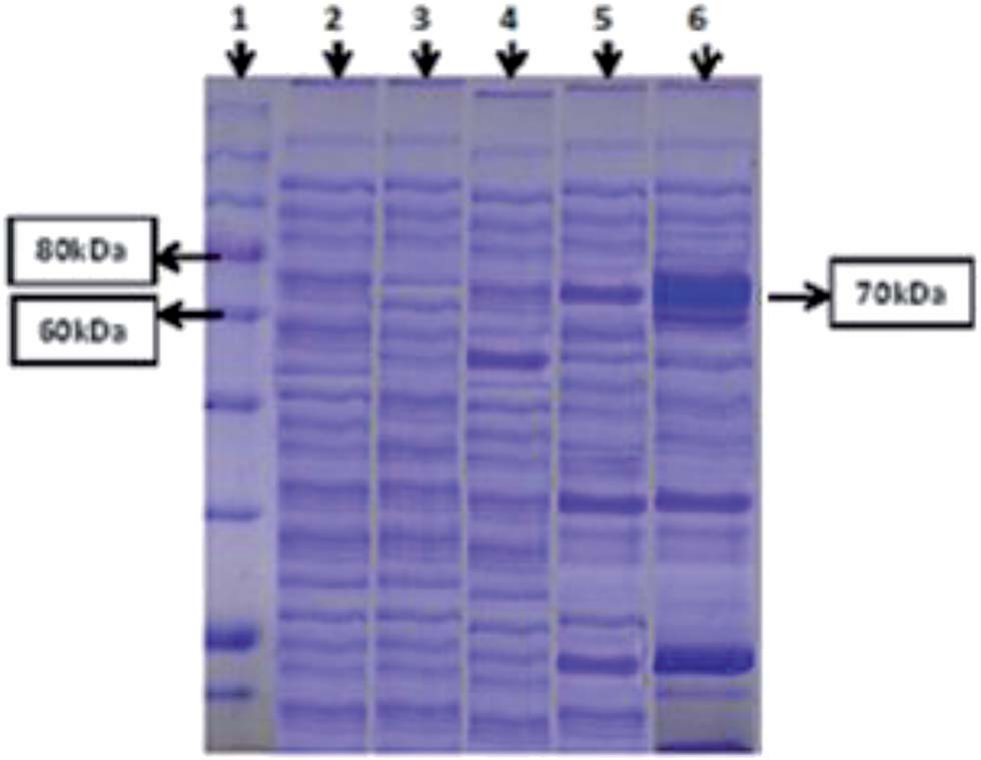
SDS-PAGE study of recombinant amylase gene expression. Protein marker (Lane 1), extract of wild *E. coli* (Lane 2), vector only without insert (Lane 3), vector plus amylase gene, non-induced (Lane 4), vector plus amylase gene, induced, extracellular fraction (Lane 5), vector plus amylase gene, induced, intracellular fraction (Lane 6). The results are for 70 kDa α-amylase in the intracellular fraction.

Both intracellular as well as extracellular enzyme samples were analyzed for amylase activity. To obtain the intracellular enzyme fraction, the cultivated bacterial cells were subjected to sonication for lyses in a heat sonicator system. In the intracellular fraction, 2.5 U ml^−1^ min^−1^ of amylase activity was found. However, minute activity of the amylase enzyme was observed in the extracellular samples. SDS-PAGE analysis was also done for both (extracellular and intracellular fractions) samples to ensure the successful expression of the amylase gene of *T. petrophila* into *E. coli* BL21 (DE3). A protein marker, positive (BL21 containing pET 21a (+) without the gene) and negative controls (wild BL21 DE3) were also run in parallel. A band of almost 70 kDa was found in the intracellular enzyme fraction, as shown in Fig. 2, while no such band was observed in the positive and negative controls at the same position. However, a very light protein band was seen at the same position in the extracellular enzyme fraction (Fig. 2).

### Optimization of the parameters for α-amylase production

This study was concerned with the scale-up studies of cloned α-amylase from *Thermotoga petrophila* for high production by optimization of different parameters and evaluation of the recombinant amylase for its desizing potential. These studies were carried out in a 7.5 L glass fermenter fitted with a 5 L round-bottom flask. For high recombinant α-amylase production, the following parameters were optimized during the fermenter studies.

### Influence of the agitation rate

**Fig. 3:**
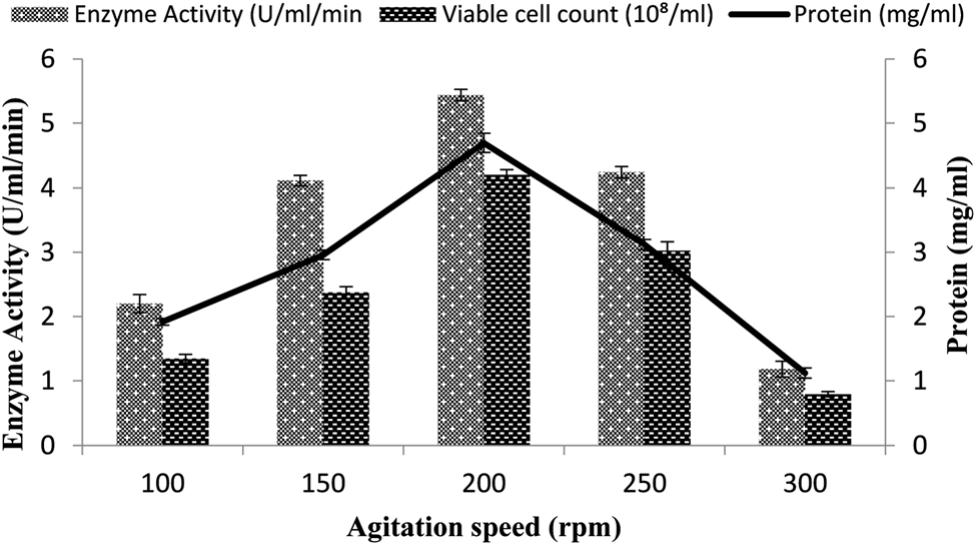
Effect of the rate of agitation on amylase activity in the stirred fermenter at an initial temperature of 22 °C and pH of 7.0.

A continuous supply of oxygen to the microorganism in the fermentation medium depends on the agitation intensity. To determine the effect of agitation on α-amylase production in the stirred fermenter, a 100–300 rpm agitation speed was utilized together with a 3% inoculum size with 20% dissolved oxygen level, 60% volume of medium, and 3 vvm aeration rate. The highest enzyme activity (5.43 ± 0.08 U ml^−1^ min^−1^) was obtained at 200 rpm with 4.69 ± 0.14 mg ml^−1^ total protein (Fig. 3). A further increase in the agitation intensity resulted in decreased enzyme production, mainly due to the oxidative stress and disproportionate foaming to the organism. A low agitation intensity also had a marked impact on enzyme production due to the limited oxygen supply to the bacterial cells and poor mixing of the medium for fermentation. At 100 and 150 rpm, the enzyme activity was calculated to be 2.2 ± 0.14 U ml^−1^ min^−1^ and 4.114 ± 0.08 U ml^−1^ min^−1^ and the total protein was 1.916 ± 0.05 mg ml^−1^ and 2.958 ± 0.07 mg ml^−1^ (Fig. 3). At other agitation rates of 100, 150, 250, and 300 rpm, enzyme expression was calculated as 4.114 ± 0.08 U ml^−1^ min^−1^, 2.2 ± 0.14 U ml^−1^ min^−1^, 1.180 ± 0.12 U ml^−1^ min^−1^, and 4.243 ± 0.08 U ml^−1^ min^−1^, while the total protein contents were 1.1198 ± 0.07 mg ml^−1^, 1.916 ± 0.05 mg ml^−1^, 3.12 ± 0.07 mg ml^−1^, and 2.958 ± 0.07 mg ml^−1^, respectively. The total viable cell counts were 1.34 × 10^8^/ml, 2.37 × 10^8^/ml, 4.2 × 10^8^/ml, 3.0 × 10^8^/ml, and 0.79 × 10^8^/ml at 100, 150, 200, 250, and 300 rpm, respectively (Fig. 3). However, Haq *et al.*^35^ in their fermenter studies reported the maximum amylase production from *Bacillus amyloliquefaciens* at 400 rpm.

### Effect of the inoculum size

**Fig. 4:**
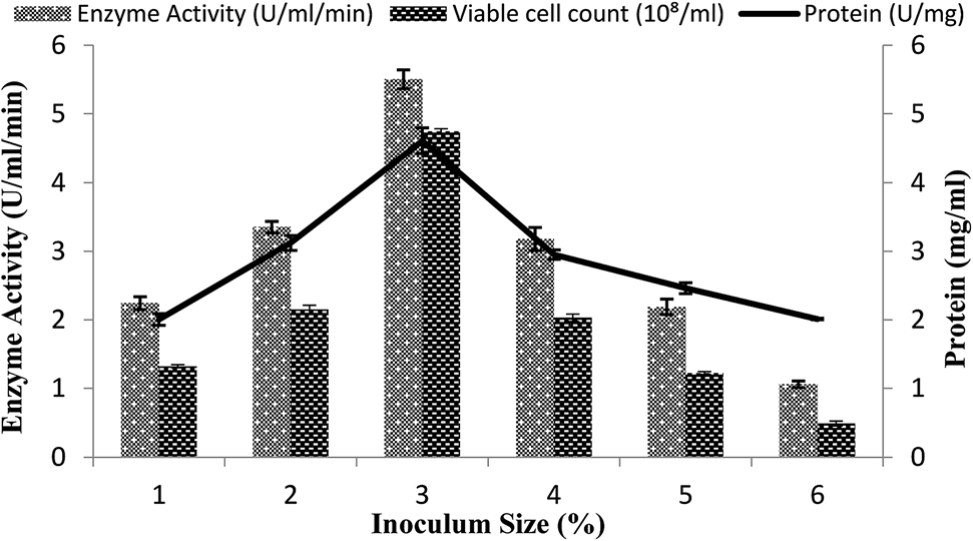
Effect of the inoculum size on amylase activity in the stirred fermenter at an initial pH of 7.0 and temperature of 22 °C.

The size of the inoculums is also an important factor in the fermentation reactions.^40^ A range of inoculum sizes (1–6%) that yielded viable cells (2.2 × 10^8^ to 3.45 × 10^8^ ml^−1^) were utilized for the optimization process in the stirred fermenter. The fermenter medium was inoculated with varying percentages (1–6%) of inoculums, with setting the conditions as a 200 rpm agitation rate, 20% dissolved oxygen level, 60%, volume of the medium, and 3 vvm aeration rate. The maximal enzyme activity obtained was 5.50 ± 0.13 U ml^−1^ min^−1^ with 3% inoculum having 4.61 ± 0.18 mg ml^−1^ total protein content and 4.74 × 10^8^/ml total viable cell count as shown in Fig. 4. A decline in enzyme activity was observed when a higher % age of inoculum was used. Similarly, α-amylase production was reduced when less than 3% inoculum was added. The α-amylase activity was found to be 2.24 ± 0.09 U ml^−1^ min^−1^ and 3.35 ± 0.08 U ml^−1^ min^−1^ with a total protein content of 2.0 ± 0.08 mg ml^−1^ and 3.12 ± 0.10 mg ml^−1^ and viable cell count of 1.32 × 10^8^/ml and 2.15 × 10^8^/ml when 1% and 2% inoculum was added, respectively (Fig. 4). This result revealed that a lower inoculum size yielded an inadequate microorganism growth, which resulted in competitive inhibition,^41^ whereas more than 3% inoculum resulted in increased microorganism growth, which resulted in depletion of the nutrients and in turn reduction of the metabolic activity^42^ and the accretion of other byproducts in the medium.^35^

### Effect of dissolved oxygen

**Fig. 5:**
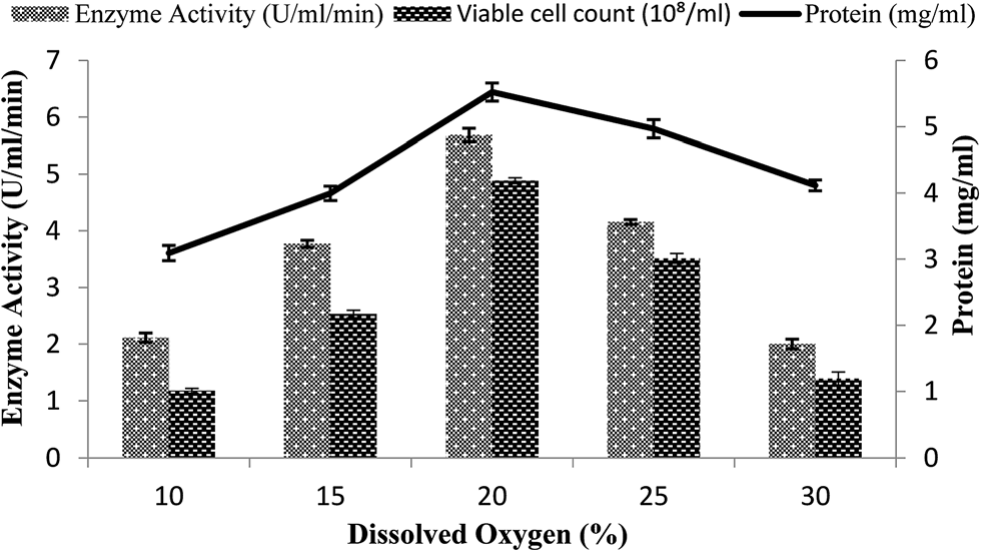
Effect of dissolved oxygen on the amylase activity in the stirred fermenter at an initial pH of 7.0 and temperature of 22 °C.

A continuous supply of oxygen is necessary for aerobic fermentation by the organism present in the dissolved form in the medium. Various dissolved oxygen levels ranging from 10–30% were analyzed by providing 3% inoculum at 200 rpm agitation speed and 3 vvm aeration rate and by setting the volume of the medium as 60% in order to achieve the maximum enzyme production. In the present study, the maximum amylase production (5.68 U ml^−1^ min^−1^) was obtained when 20% dissolved oxygen was present in the medium, as shown in Fig. 5. Low and high concentrations of oxygen levels might have a detrimental effect on the microorganisms and may retard their growth.^43^ At high concentration, oxygen may cross the saturation level, leading to the formation of many toxic compounds, like superoxide and hydrogen peroxide, in the fermentation media.^44–46^ At the other 10%, 15%, 25%, and 30% levels of dissolved oxygen, low enzyme activity was obtained (2.11 ± 0.08 U ml^−1^ min^−1^, 3.77 ± 0.06 U ml^−1^ min^−1^, 4.15 ± 0.04 U ml^−1^ min^−1^, and 2.0 ± 0.08 U ml^−1^ min^−1^) and less total protein content was obtained (4.11 ± 0.08 mg ml^−1^, 3.09 ± 0.11 mg ml^−1^, 4.96 ± 0.13 mg ml^−1^, and 3.99 ± 0.1 mg ml^−1^) and viable cell count (2.53 × 10^8^/ml, 1.17 × 10^8^/ml, 1.39 × 10^8^/ml, and 3.51 × 10^8^/ml) were achieved (Fig. 5). Abdullah *et al.*^22^ claimed increased amylase production from *Aspergillus oryzae* in a stirred fermenter with 15% dissolved oxygen.

### Effect of fermentation medium volume

**Fig. 6:**
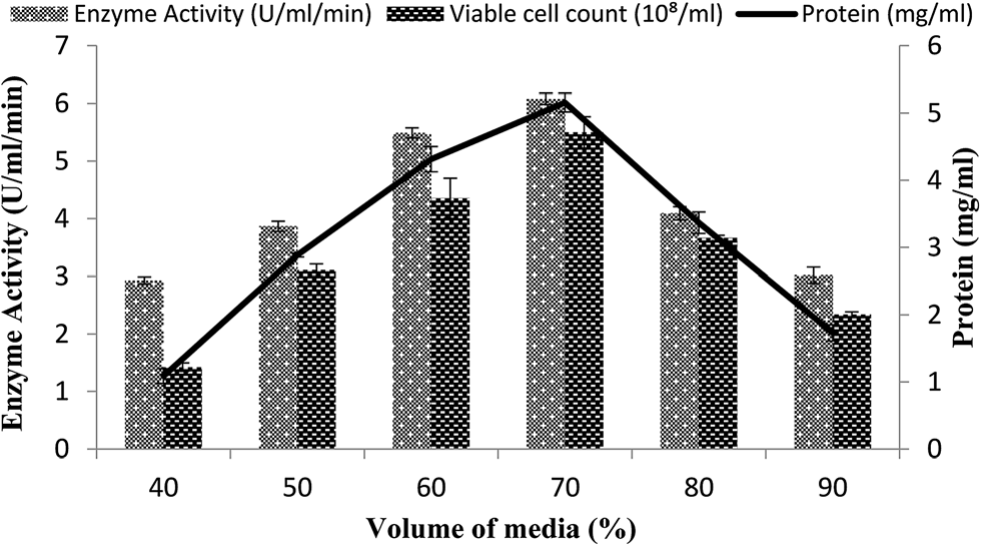
Effect of the medium volume on the activity of amylase in a stirred fermenter at an initial pH 7.0 and temperature of 22 °C.

The volume of the fermentation medium exhibited an important role during the production of enzymes as in the case for *Bacillus* sp. using submerged fermentation.^47^ Aeration and agitation affected the enzyme production and also affected the growth of the organisms. To determine the effect of the volume of fermentation medium for maximum enzyme production, different volumes 40–90% of the fermentation medium were used in a stirred fermenter, while setting the other condition as 3% inoculum size, 200 rpm agitation speed, 20% dissolved oxygen level, and 3 vvm aeration rate. The maximum enzyme production (6.07 U ml^−1^ min^−1^) with 70% volume of medium suggested that, at increased levels of medium volume, the growth of microorganisms was restricted as the supply of oxygen decreased and also as the availability of nutrients decreased due to the inadequate agitation.^48^ Total calculated protein content was 5.15 ± 0.13 mg ml^−1^ and viable cell count was 5.5 × 10^8^/ml when utilizing a working volume of 70%, as shown in Fig. 6. A reasonable amount of enzyme activity (5.49 ± 0.08 U ml^−1^ min^−1^) with a total protein content of 4.31 ± 0.18 mg ml^−1^ and viable cell count of 4.35 × 10^8^/ml was also obtained when a 60% working volume was used (Fig. 6). Very less enzyme activity, *i.e.*, 2.92 ± 0.06 U ml^−1^ min^−1^ and 3.01 ± 0.13 U ml^−1^ min^−1^ with a total protein content of 1.08 ± 0.1 mg ml^−1^ and 1.73 ± 0.12 mg ml^−1^, was observed when 40% and 90% working volumes were utilized while the viable cell counts were 1.42 × 10^8^/ml and 2.34 × 10^8^/ml, respectively.

### Effect of rate of aeration

**Fig. 7:**
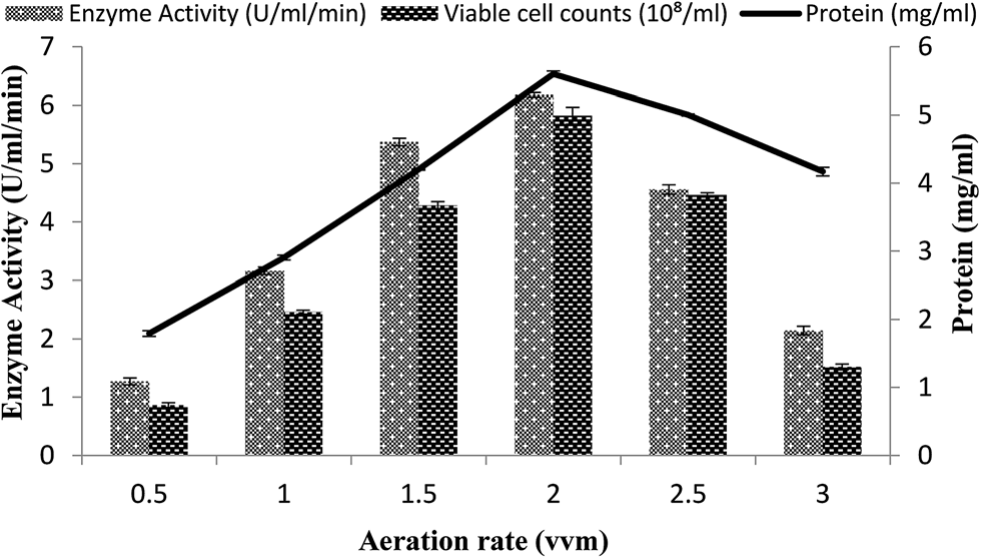
Effect of the rate of aeration on amylase activity in the stirred fermenter at an initial temperature of 22 °C and pH 7.0.

The effect of the rate of aeration on the production of recombinant α-amylase was studied by different aeration levels (0.5–3.0 vvm), with setting the other conditions as 3% inoculum size, 200 rpm agitation rate, 20% dissolved oxygen level, and 70% volume of the medium. The maximum enzyme activity (6.17 ± 0.03 U ml^−1^ min^−1^) was observed at an aeration level of 2.0 vvm with 5.59 ± 0.04 mg ml^−1^ total protein content and a viable cell count of 5.82 × 10^8^/ml (Fig. 7). At aeration rates of 0.5, 1.0, 1.5, 2.5, and 3.0 vvm, the enzyme activity was 3.16 ± 0.06 U ml^−1^ min^−1^, 2.10 ± 0.02 U ml^−1^ min^−1^, 1.27 ± 0.06 U ml^−1^ min^−1^, 4.55 ± 0.08 U ml^−1^ min^−1^, and 5.37 ± 0.06 U ml^−1^ min^−1^, with total protein content of 4.2008 ± 0.0103 mg ml^−1^, 2.904 ± 0.0354 mg ml^−1^, 4.169 ± 0.063 mg ml^−1^, 1.794 ± 0.04 mg ml^−1^, 5.001 ± 0.0151 mg ml^−1^, and viable cell count of 1.51 × 10^8^/ml, 2.45 × 10^8^/ml, 4.46 × 10^8^/ml, 4.28 × 10^8^/ml, and 0.86 × 10^8^/ml, respectively (Fig. 7). It has been reported that higher aeration rates have an adverse effect on the enzyme production and at low levels due to the improper supply of oxygen, which adversely hamper the growth of the organism.^49^

### Media optimization with IPTG and lactose as the inducers

**Fig. 8:**
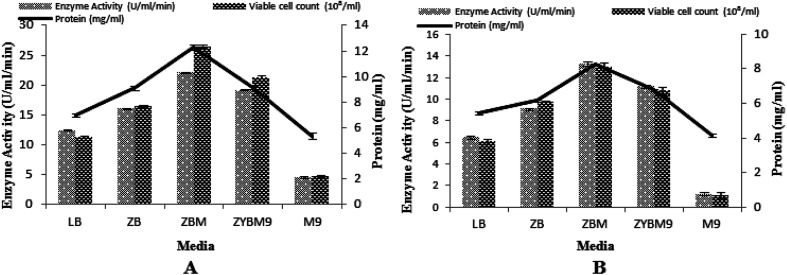
(A) Effect of different media induced with lactose on the enzyme activity. (B) Effect of different media induced with IPTG on the enzyme activity.

In this study, the T7-regulated pET 21a (+) vector was used. To determine the transcription efficiency of the T7 promoter, two inducers, namely IPTG and lactose, were used. This is an important study by IPTG with its low cost analog, lactose, which was used as an inducing agent and produced encouraging results.^50^ Being a natural inducer of lac operon, lactose has proved to be non-toxic to microorganisms and also utilized by microorganisms as a carbon source.^17,51^ Five different media were tested to determine the maximum induction of T7 promoter for α-amylase production, with setting 3% inoculum, 200 rpm agitation speed, 2 vvm aeration rate, 20% dissolved oxygen level, and 70% volume of the medium. The maximum production of α-amylase (22.08 ± 0.11 U ml^−1^ min^−1^) was obtained with 12.27 ± 0.19 mg ml^−1^ total protein content when the T7 promoter of pET 21a (+) was induced with lactose and recombinant *E. coli* was grown in ZBM medium. Similarly, a better yield of α-amylase (13.28 ± 8.26 U ml^−1^ min^−1^) with 8.26 ± 0.04 mg ml^−1^ total protein content was obtained when IPTG was used as an inducer in ZBM medium. This ZBM medium is a modified form of ZB medium^52^ containing tryptone, the amino acid source, more than other tested media.^53^ A lower yield of enzyme was produced in M9 medium either induced with IPTG or lactose. Generally, induction with lactose produced better yields in all the media used as compared to induction by IPTG. The total viable cell count in lactose-induced media was 26.49 × 10^8^/ml, 21.73 × 10^8^/ml, 16.46 × 10^8^/ml, 11.34 × 10^8^/ml, and 4.74 × 10^8^/ml among ZBM, ZYBM9, ZB, LB, and M9, respectively. While in IPTG-induced media, the total viable cell count was 12.93 × 10^8^/ml, 10.78 × 10^8^/ml, 9.68 × 10^8^/ml, 6.09 × 10^8^/ml, and 1.08 × 10^8^/ml among ZBM, ZYBM9, ZB, LB, and M9, respectively (Fig. 8A and B).

### Optimization of the desizing potential of α-amylase

**Fig. 9:**
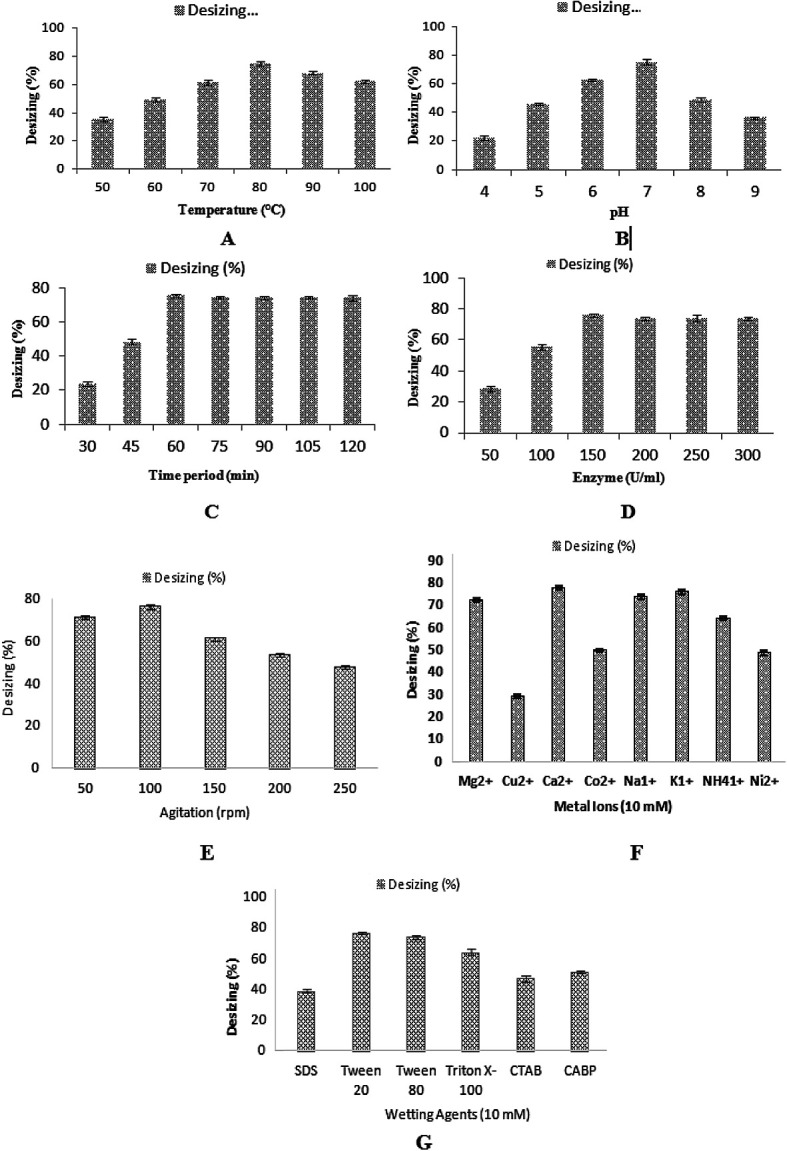
(A) Effect of incubating temperature on the desizing of cotton cloth. (B) Effect of pH on the desizing of cotton cloth. (C) Effect of time of incubation on the desizing of cotton cloth. (D) Effect of enzyme concentration on the desizing of cotton cloth. (E) Effect of agitation speed on the desizing of cotton cloth. (F) Effect of metal ions on the desizing of cotton cloth. (G) Effect of wetting agents on the desizing of cotton cloth.

In the textile industry, the use of thermostable amylase is very auspicious for the desizing of cotton cloth. The sized fabric is actually hydrophobic in nature and the wet processing, which includes desizing and scouring, is an attempt to make the fabric hydrophilic in nature. Although, the sized fabric would not become completely hydrophilic after the desizing process, after processing, like scouring, the fabric can become completely hydrophilic in nature. In the present study, the desizing potential of this recombinant thermostable amylase was optimized for maximum desizing activity. The effects of temperature, pH, different concentrations of enzyme and time of incubation were checked for the desizing of cotton cloth. A plain weaved 100% cotton grey fabric was used in this experiment. The alignment of the warps and wefts in the plain weaved fabric was present in a simple crisscross pattern,^31^ which is used more than any other weave. Plain weaved fabric is stronger than any other weave type due to the presence of a greater number of intersections per unit space, all other factors being equal.^54^ A fabric piece (5 × 5 inches) was pre-weighed. The pre-weight of the cloth having starch on it was calculated as 13.78 g. The total fiber count was 120 epi in the grey fabric used in this study. The cotton cloth of size (5 × 5 inches) was dipped in 100 ml of α-amylase and incubated at various temperatures ranging from 50 °C to 100 °C for 60 min. The maximum desizing percentage for cotton cloth was obtained (74.8%) at 80 °C when incubated for 1 h (Fig. 9A). However, 68.43% and 62.57% desizing was obtained at 90 °C and 100 °C. The effect of pH was determined on the desizing of the cotton cloth (5 × 5 inches) by incubating it in enzyme of different pH of buffer (pH 4–9). At pH 7.0, the enzyme maximum desizing of cotton cloth (75.03%) was obtained, while decreased desizings of cotton cloth (45.80%, 62.84%, 49.12%, and 36.22%) were achieved when the pH of the enzyme was adjusted to 5, 6, 8, and 9, respectively (Fig. 9B). The effect of the time of incubation was calculated by treating the cotton cloth (5 × 5 inches) with enzyme for 30–120 min at 80 °C. The maximum desizing (75.349%) of the cotton cloth was achieved after incubating it for 60 min (Fig. 9C). Further increasing the time duration did not affect the rate of desizing, with values of 74.24%, 74.09%, 74.33%, and 74.16% desizing obtained after 75, 90, 105, and 120 min of incubation respectively. To determine the effect of enzyme concentration on the desizing of the cotton cloth (5 × 5 inches), different enzyme concentrations (50–300 U ml^−1^) were used. The cloth was incubated with different concentrations of enzyme with pH 7.0 at 80 °C for 60 min. With the enzyme concentration of 150 U ml^−1^, the maximum desizing percentage (75.88%) was obtained (Fig. 9D). Increasing the concentration of enzyme from this optimal level seemed to inhibit the desizing activity, which might be due to the inappropriate ratio of enzyme to the substrate.^54^ The effect of the agitation rate on the desizing of the cotton cloth by recombinant enzyme was investigated by incubating the cloth with 150 U ml^−1^ of enzyme at 80 °C and pH 7.0 for 60 min at various agitation speeds (50–250 rpm). The maximum desizing (76.35%) was achieved at 100 rpm agitation speed, as shown in Fig. 9E. A higher agitation rates resulted in decreased desizing percentages, which might be due to the improper interaction of enzyme with its substrate. Almost 61.16%, 53.46%, and 47.74% desizing was observed at 150 rpm, 200 rpm, and 250 rpm, respectively. Similarly, 71.2% desizing was observed at 50 rpm as shown in Fig. 9E. Various metal ions, including Mg^2+^, Cu^2+^, Ca^2+^, Co^2+^, Na^1+^, K^1+^, NH_4_^1+^, and Ni^2+^ were analyzed to determine their effect on the desizing activity of recombinant α-amylase enzymes. Most of the metal ions did not show any considerable effect on the activity of enzyme, while in the presence of 10 mM Ca^2+^ the increased desizing activity (77.7%) was observed, as shown in Fig. 9F. In the presence of Mg^2+^, Cu^2+^, Co^2+^, Na^1+^, K^1+^, NH_4_^1+^, and Ni^2+^, the desizing activity was 72%, 29%, 49%, 73%, 75%, 64%, and 48%, respectively, as shown in Fig. 9F. Different ionic, nonionic, and amphoteric wetting agents, including SDS, Tween 20, Tween 80, Triton X-100, CTAB, and CABP, were used in 10 mM concentration to determine their effect on the desizing potential of recombinant α-amylase enzyme. The results obtained showed that α-amylase retained 76%, 73%, and 64% desizing activity in the presence of Tween 20, Tween 80, and Triton X-100, respectively, as shown in Fig. 9G. However, ionic and amphoteric wetting agents, including SDS, CTAB, and CABP, reduced the desizing activity of α-amylase to 38%, 47%, and 51%, respectively. This reduction in activity might have resulted due to the distortion in the enzyme–substrate complex as well as due to change in electrostatic potential of the substrate due to the adsorption of ionic wetting agents.^55,56^

### Assessment of the desizing effectiveness by α-amylase enzyme

**Fig. 10:**
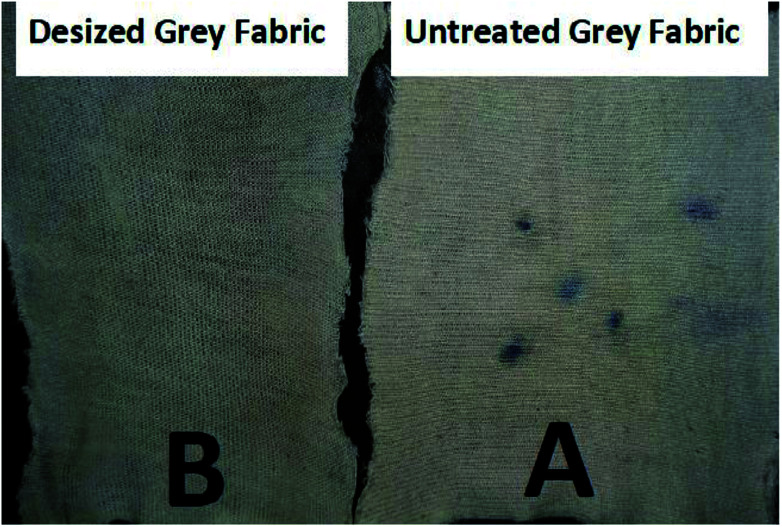
Assessment of the desizing potential of recombinant α-amylase enzyme on grey fabric: (A) the spots present on untreated grey fabric are purple, (B) no spot on the desized fabric.

To assess the effectiveness of the desizing efficiency of grey fabric by recombinant α-amylase enzyme, the iodine solution test was used before and after desizing of the grey fabric. The results obtained after the iodine test are represented in Fig. 10. A very clear purplish spot appeared following the application of iodine solution on untreated grey fabric, while after desizing by the enzyme no such purple color appeared on the grey fabric. These results confirmed that starch present as a sizing agent in the grey fabric was completely hydrolyzed by the application of the recombinant α-amylase enzyme.

## Research involving human participants and/or animals

Authors claimed that the present research work does not include any human or animal study.

## Informed consent

Authors included in the study showed willingness to submit research work in the journal and informed consent was obtained from all individuals.

## Funding

Funding for this project was provided by Higher Education Commission, Pakistan as PhD research work for Dr Asma Zafar.

## Conflicts of interest

The authors declare that they have no conflict of interest.

## Supplementary Material
